# Adipose-Derived Mesenchymal Stem Cells-Derived Exosomes Carry MicroRNA-671 to Alleviate Myocardial Infarction Through Inactivating the TGFBR2/Smad2 Axis

**DOI:** 10.1007/s10753-021-01460-9

**Published:** 2021-04-21

**Authors:** Xue Wang, Yuhai Zhu, Chengcheng Wu, Wennan Liu, Yujie He, Qing Yang

**Affiliations:** 1 Department of Cardiology, Tianjin Medical University General Hospital, NO. 154, Anshan Street, Heping District, Tianjin, 300052 People’s Republic of China; 2 Department of Medical Cosmetology, Tianjin Medical University General Hospital Airport Hospital, Tianjin, 300308 People’s Republic of China; 3 Department of Cardiology, Tianjin Beichen District Chinese Medicine Hospital, Tianjin, 300400 People’s Republic of China

**Keywords:** myocardial infarction, MSC, exosomes, microRNA-671, TGFBR2, Smad2

## Abstract

Mesenchymal stem cells (MSCs) and their derived extracellular vesicles have been reported as promising tools for the management of heart disease. The aim of this study was to explore the function of adipose-derived MSCs (adMSCs)-derived exosomes (Exo) in the progression of myocardial infarction (MI) and the molecules involved. Mouse cardiomyocytes were treated with oxygen-glucose deprivation (OGD) to mimic an MI condition *in vitro*. The adMSCs-derived Exo were identified and administrated into the OGD-treated cardiomyocytes, and then the viability and apoptosis of cells, and the secretion of fibrosis- and inflammation-related cytokines in cells were determined. Differentially expressed microRNAs (miRNAs) in cells after Exo treatment were screened using a microarray analysis. The downstream molecules regulated by miR-671 were explored through bioinformatic analysis. Involvements of miR-671 and transforming growth factor beta receptor 2 (TGFBR2) in the exosome-mediated events were confirmed by rescue experiments. A murine model with MI was induced and treated with Exo for functional experiments *in vivo*. Compared to phosphate-buffered saline treatment, the Exo treatment significantly enhanced viability while reduced apoptosis of cardiomyocytes, and in reduced myocardial fibrosis and inflammation both *in vitro* and *in vivo*. miR-671 was significantly upregulated in cells after Exo treatment. Downregulation of miR-671 blocked the protective functions of Exo. miR-671 targeted TGFBR2 and suppressed phosphorylation of Smad2. Artificial downregulation of TGFBR2 enhanced viability of the OGD-treated cardiomyocytes. This study suggested that adMSC-derived exosomal miR-671 directly targets TGFBR2 and reduces Smad2 phosphorylation to alleviate MI-like symptoms both *in vivo* and *in vitro*.

## INTRODUCTION

Acute myocardial infarction (MI) is defined as the acute myocardial injury with the clinical appearance of acute myocardial ischemia along with a rise and/or fall of cardiac troponin values [1, 2]. MI is commonly known as “heart attack,” which induces the formation of noncontractile scar and the remodeling of left ventricular (LV) that further deteriorate cardiac function [3]. The consequential ischemic heart disease is a leading cause of disability and death worldwide [4]. Accumulated LV mass and progressive thickening of the myocardium are typical indicators of LV remodeling and the associated cardiac hypertrophy, apoptosis, and fibrosis [3]. The current clinical treatments for heart failure following MI are instrument implantation and drug use; however, the efficacy remains unsatisfactory [5]. In addition, the surgical interventions and thrombolysis may induce secondary reperfusion injury, leading to further irreversible death of cardiomyocytes [6]. Developing less-harmful and effective alternatives for MI management while limiting cardiomyocyte loss is of great significance.

Upon MI, cardiomyocyte death induces robust inflammatory response which evolves cardiac injury, repair, and remodeling. Exosomes have been increasingly recognized to be implicated in immune regulation and inflammatory response following MI [7]. Exosomes are cell-sourced nanosized vesicles containing molecular substances including microRNAs (miRNAs), lipids, proteins, and nucleic acids and are used as a major type of mediators responsible for intercellular communications in different pathological conditions including ischemic heart disease [8]. Mesenchymal stem cells (MSCs) are one of the most ideal cell therapy tools due to their advantages in easy-isolation, self-renewal, and expansion potentials, and the adipose-derived MSCs (adMSCs) and their released exosomes have seen advantages in clinical application [9]. miRNAs are frequently involved and also play critical functions in exosome-mediated events [10]. They are a major class of highly conserved non-coding RNAs involved in a wide array of biological events and cardiac diseases such as MI, heart failure, and cardiomyocyte hypertrophy due to their potent regulation on diverse target genes [11]. Here, our miRNA microarray analysis suggested that miR-671 was significantly upregulated in cells after treatment with adMSCs-derived exosomes (hereafter termed Exo; only indicating the exosomes derived from the adMSCs in this study). miR-671 has been reported to be poorly expressed in patients with coronary artery disease [12]. Whether exosomal miR-671 exerts functions in myocardial protection remains unknown. The subsequent integrated analysis suggested transforming growth factor beta (TGF-β) receptor 2 (TGFBR2) as a candidate target gene of miR-671. The TGF-β1 signal transduction pathway has been well-recognized to play a crucial role in the process of myocardial fibrosis [13]. Smad proteins, the intracellular effectors of TGF-β signaling, are activated by the TGFBRs and translocate into nucleus where they regulate transcription activities [14]. Smad2 and Smad3 are frequently involved in TGF-β1-mediated pro-fibrotic events [15, 16]. TGFBR2 has been reported as an activator of Smad2 phosphorylation [17, 18]. In this study, we hypothesized that Exo contain miR-671 and alleviate MI symptoms through suppression of the TGFBR2/Smad2 axis. Acquired adMSCs were used for Exo extraction, and gain- and loss-of-function studies were performed both *in vivo* and *in vitro* to validate the hypothesis.

## MATERIALS AND METHODS

### Culture and Identification of Cells

Mouse adMSCs (CP-M138) and mouse cardiomyocytes (CP-M073) were procured from Procell Life Science & Technology Co., Ltd. (Wuhan, Hubei, China). The cells were cultured in adMSC-specific medium (CM-M138) and cardiomyocyte-specific medium (CM-M073) in a 37 °C incubator with 5% CO_2_.

To identify the adMSCs, the phenotypic profile of cells was examined by flow cytometry using phycoerythrin-labeled human anti-CD29 (#102216, Biolegend, San Diego, CA, USA), anti-CD44 (#338804, Biolegend), anti-CD90 (#328109, Biolegend), anti-CD45 (#103106, Biolegend), and anti-immunoglobulin G (IgG, Biolegend). The differentiation potential of adMSCs was examined by osteogenic differentiation (CP1206) and adipogenic differentiation (CP1215) kits (Weitong Biotechnology, Shenzhen, Guangdong, China) using alizarin red staining and Oil red O staining, respectively.

### Cell Transfection

The adMSCs were allocated into adMSC group (without transfection, and the corresponding exosomes were named Exo), NC-inhibitor group (adMSCs were transfected with NC inhibitor, and the exosomes were named Exo-NC, NC refers to negative control), miR-671 inhibitor group (adMSCs were transfected with miR-671 inhibitor, and the exosomes were named Exo-inhibitor).

The cardiomyocytes were allocated into the following groups after corresponding transfection: Control group, NC mimic group (cardiomyocytes were transfected with NC mimic), miR-671 mimic (cardiomyocytes were transfected with miR-671 mimic), oxygen-glucose deprivation (OGD) group (cardiomyocytes underwent OGD treatment), phosphate-buffered saline (PBS) group (OGD-induced cardiomyocytes were further treated with PBS for 24 h), Exo group (OGD-induced cardiomyocytes were further treated with Exo for 24 h), Exo-NC group (OGD-induced cardiomyocytes were further treated with Exo-NC for 24 h), Exo-inhibitor group (OGD-induced cardiomyocytes were further treated with Exo-inhibitor for 24 h), Exo-inhibitor + si-NC (small interfering-NC) group (OGD-induced cardiomyocytes were treated with Exo-inhibitor for 24 h and then transfected with si-NC), and Exo-inhibitor + si-TGFBR2 group (OGD-induced cardiomyocytes were treated with Exo-inhibitor for 24 h and then transfected with si-TGFBR2). In all cellular experiments involving Exo treatment (except for the Exo uptake experiment), the cardiomyocytes were pre-treated with OGD before Exo treatment. The Exo were administrated after OGD at different time points to determine the suitable interval (see details in the result section).

The dose and duration of Exo administration in cells was 50 μg/mL for 24 h. The miR-671 mimic/inhibitor, si-TGFBR2, and the controls were acquired from GenePharma Co., Ltd. (Shanghai, China). All transfections were performed using the Lipofectamine™ 2000 transfection reagent (Thermo Fisher Scientific Inc., Waltham, MA, USA).

### OGD-Treated Cardiomyocytes for *In Vitro* Study

The cardiomyocytes were cultured in glucose-free Dulbecco’s modified Eagle’s medium (Thermo Fisher Scientific) in an anaerobic incubator (1% O_2_, 5% CO_2_ and 94% N_2_) at 37 °C for 0 h, 6 h, 12 h, 18 h, and 24 h, respectively. The control cells were cultured under normoxic condition.

### Isolation and Identification of Exo

Exo were extracted from the culture medium of different adMSCs using Total Exosome Isolation Reagent (#4478359, Thermo Fisher Scientific) as previously reported [19]. The adMSC-specific medium was first ultra-centrifuged at 100,000×*g* overnight to exhaust the original exosomes in the medium, and the adMSCs were cultured in the medium for 48 h. After that, the culture medium was centrifuged at 2,000×*g* for 30 min to discard cell debris. Then, the supernatant was loaded in a new tube and mixed with isolation reagent (v:v = 2:1). The mixture was cultured at 4 °C overnight and then centrifuged at 10,000×*g* at 4 °C for 1 h. The Exo spheres were resuspended in PBS for further use.

Then, the ultrastructure of the Exo was observed under a transmission electron microscope (TEM, Libra 120; Zeiss, Oberkochen, Germany). Distribution of particle size was analyzed by nanoparticle tracking analysis (NTA) using a Nanosight LM10 (Malvern Instruments, Malvern, UK) and the NTA v.3.0 software (Malvern Instruments). Expression of exosome biomarkers CD63 (ab217345, Abcam Inc., Cambridge, MA, USA) and CD81 (ab109201, Abcam) in the particles was examined by western blot analysis.

### Uptake of Exo by the Cardiomyocytes

The extracted exosomes were labeled using a PKH26 red fluorescence kit (Sigma-Aldrich Chemical Company, St Louis, MO, USA) in accordance with the kit’s instructions. Next, the Exo were resuspended and incubated with the mouse cardiomyocytes at 37 °C for 24 h. After that, the cells were washed twice in PBS, fixed in 4% paraformaldehyde for 10 min, and stained with 4', 6-diamidino-2-phenylindole. The PKH26-labeled exosomes internalized by cardiomyocytes were observed under a confocal laser scanning microscope (Leica, Solms, Germany).

### 3-(4, 5-Dimethylthiazol-2-yl)-2, 5-Diphenyltetrazolium Bromide (MTT) Assay

Viability of cells was examined using an MTT kit (Beyotime Biotechnology Co. Ltd., Shanghai, China). In brief, the cells incubated in 96-well plates were incubated with MTT solution (10 μL per well) for 4 h. Thereafter, each well was added with 100 μL formazan solution to dissolve the formazan sediments. After 3–4 h, the violet formazan crystals were fully dissolved, and the optical density (OD) value at 570 nm was evaluated using a spectrophotometer to examine the viability of cells.

### Cell Apoptosis Detection by Flow Cytometry

An Annexin V-fluorescein isothiocyanate (FITC) cell apoptosis detection kit (Thermo Fisher Scientific) was used to evaluate cell apoptosis according to the kit’s instructions. In brief, the cells were incubated with 5 μL propidium iodide and 5 μL Annexin V-FITC at room temperature in the dark for 5 min. Thereafter, the apoptotic rate in cells was examined using a flow cytometer (FACS Verse, BD, Biosciences, Franklin Lakes, NJ, USA), and the data were analyzed by the Flow J software (Tree-star Inc, San Carlos, CA, USA).

### Reverse Transcription-Quantitative Polymerase Chain Reaction

Total RNA from cells or tissues was extracted using RNAiso Plus (Takara Holdings Inc., Kyoto, Japan) and quantified using a NanoDrop spectrophotometer (Thermo Fisher Scientific). Next, 500 ng RNA was reversely transcribed into first-strand cDNA using an M-MLV Reverse Transcriptase (Invitrogen, Thermo Fisher Scientific). After that, real-time qPCR was conducted using the VeriQuest Fast SYBR Green qPCR Master Mix (Thermo Fisher Scientific) on a StepOneplus Real-time PCR System (Applied Biosystems, Carlsbad, CA, USA). The primer sequences are listed in Table 1, in which 5s and GAPDH were used for the internal loading for miRNA and mRNA, respectively. The fold change of gene expression was calculated by 2^-ΔΔCT^ method.

**Table 1:** Primer Sequences for RT-qPCR

Gene	Primer sequence (5'-3')
miR-671	F: GGAAGCCCTGGAGGGG
	R: GAACATGTCTGCGTATCTC
TGFBR2	F: CCTACTCTGTCTGTGGATGACC
	R: GACATCCGTCTGCTTGAACGAC
NF2	F: GCTCAGGACCTGGAGATGTATG
	R: CAGCCTGTTCTCAGGGTCATAG
FBXW11	F: TGCCTCCAGTATGATGAGCGAG
	R: GTCCATTGCTGAAGCGTAAGTGC
DLG1	F: GTGAAAAGGCGGAAGCCAGCAT
	R: GTGCTGATTTCCAACACCTCCAG
DVL3	F: TGATGGACGCATTGAGCCAGGA
	R: ACAATCTCCCGAAGGACTCGGA
YWHAZ	F: CAGAAGACGGAAGGTGCTGAGA
	R: CTTTCTGGTTGCGAAGCATTGGG
GAPDH	F: CATCACTGCCACCCAGAAGACTG
	R: ATGCCAGTGAGCTTCCCGTTCAG
5s	F: CTCGCTTCGGCAGCACAT
	R: TTTGCGTGTCATCCTTGCG

*RT-qPCR*, reverse transcription quantitative polymerase chain reaction; *miR-671*, microRNA-671; *TGBFR2*, transforming growth factor beta (TGF-β) receptor 2; *NF-2*, neurofibromin 2; *FBXW11*, F-box and WD repeat domain containing 11; *DLG1*, discs large MAGUK scaffold protein 1; *DVL3*, dishevelled segment polarity protein 3; *YWHAZ*, tyrosine 3-monooxygenase/tryptophan 5-monooxygenase activation protein zeta; *GAPDH*, glyceraldehyde-3-phosphate dehydrogenase; *F*, forward; *R*, reverse

### Western Blot Analysis

Total protein from the cardiomyocytes was extracted using radio-immunoprecipitation assay lysis buffer (Beyotime), and the protein concentration was assessed utilizing a bicinchoninic acid assay kit (Thermo Fisher Scientific). An equal volume of protein sample (50 μg) was separated by 12% sodium dodecyl sulfate-polyacrylamide gel electrophoresis and transferred to polyvinylidene fluoride membranes. The membranes were blocked in 5% bovine serum albumin at 20 °C for 2 h and incubated with the primary antibodies against B-cell lymphoma-2 (Bcl-2, 1:1,000, #3498, Cell Signaling Technologies (CST), Beverly, MA, USA), Bcl-2-associated X (Bax, 1:1,000, #2772, CST), α-smooth muscle actin (α-SMA, 1:1,000, #19245, CST), TGFBR2 (1:1,000, ab269279, Abcam), Smad2 (1:1,000, ab63356, Abcam), p-Smad2 (phospho S255, 1:500, ab188334, Abcam), and GAPDH (1:10,000, ab181603, Abcam) at 4 °C overnight. Further, the membranes were incubated with secondary antibodies against goat anti-mouse IgG H&L (HRP) (1:10,000, ab205719, Abcam) or goat anti-rabbit IgG H&L (HRP) (1:10,000, ab205718, Abcam). The protein bands were developed using a BeyoECL Plus kit (Beyotime). Relative gene expression was detected using the Image J software (version 1.8.0, NIH) with GAPDH as the internal loading protein.

### Enzyme-Linked Immunosorbent Assay

The concentrations of inflammatory cytokines interleukin-6 (IL-6) and tumor necrosis factor-α (TNF-α) in cardiomyocytes and tissue homogenate of mice were determined using a mouse IL-6 enzyme-linked immunosorbent assay (ELISA) kit (ab100713, Abcam) and a mouse TNF-α ELISA kit (H052, Jiancheng Bioengineering Institute, Nanjing, Jiangsu, China) according to the instructions of kits.

### MiRNA Microarray Analysis

Differentially expressed miRNAs in cardiomyocytes after Exo treatment were screened using a miRNA microarray analysis performed by OE Biotech Company (Shanghai, China). Three samples from each group (OGD and Exo groups) were used. The samples were hybridized according the instructions of the Agilent-Mouse miRNA microarray 21.0 (8 * 60 K). The data from the raw microarray image were extracted using the Feature Extraction Software (Version 10.5, Agilent Technologies, Palo Alto, CA, USA) according the protocol of miRNA_105_Dec08 FE. After that, the data were visualized and analyzed using the GeneSpring GX Software (Version 10.0, Agilent).

### Dual-Luciferase Reporter Gene Assay

The putative binding sequence between miR-671 and TGFBR2 3’UTR was first predicted on a bioinformatic system StarBase (http://starbase.sysu.edu.cn/). Then, the wild-type (WT) TGFBR2 3’UTR sequence containing the putative binding site with miR-671 and the corresponding mutant-type (MT) TGFBR2 sequence were cloned into the pmirGLO vector (Promega, Madison, WI, USA) to construct TGFBR2-WT and TGFBR2-MT luciferase reporter vectors. Thereafter, these reporters were co-administrated with miR-671 mimic or NC mimic into 293T cells (ATCC, Manassas, VA, USA) and cultured for 48 h. After that, the luciferase activity in cells was examined using a dual-luciferase reporter gene system (Promega).

### RNA-Binding Protein Immunoprecipitation Assay

An RNA-binding protein immunoprecipitation (RIP) assay was performed according to the instructions of an EZ-Magna RIP kit (Millipore Corp., Billerica, MA, USA). In brief, cardiomyocytes at an 80–90% confluence were lysed in RIP lysis buffer. Next, the cells lysates were co-incubated with magnetic beads conjugated with human anti-Ago2 (Millipore) or control anti-IgG (Millipore). After that, the samples were incubated with Proteinase K and the immunoprecipitated RNA was collected. Relative abundancy of miR-671 and TGFBR2 was then examined by RT-qPCR.

### Establishment of a Murine Model with MI

Six- to eight-week-old C57BL/6JNifdc mice procured from Vital River Laboratory Animal Technology Co., Ltd. (Beijing, China) were housed in standard animal rooms in 12-12 h lights on-off cycle with free access to feed and water. After 1 week of acclimation, the mice were divided into following groups: sham group, MI group (model mice with MI), Exo-NC group (MI model mice were further injected with Exo-NC), and Exo-inhibitor group (MI model mice were further injected with Exo-inhibitor), 8 in each group.

MI in mouse was induced as previously reported [20]. The animals were first anaesthetized through an intraperitoneal injection (i.p) of 100 mg/kg ketamine and 10 mg/kg xylazine. Then, MI in mouse was induced through a permanent ligation of the left anterior descending (LAD) artery using a 6-0 suture. Sham operation was performed in a similar manner except for permanent ligation. In the subsequent procedures, 100 μg Exo-NC or Exo-inhibitor was dissolved in PBS and injected in the boundary area of the infarcted cardiac nearby the ligation site [21].

### Histological Examination

Four weeks after MI induction, the animals were euthanized through an overdose of pentobarbital sodium (150 mg/kg, i.p). Then, the heart was crosscut, and a part of the myocardial tissues was used for the measurement of inflammatory cytokine production and gene expression, while the other part of tissues was used for histological examination.

The myocardial tissues were fixed in 4% paraformaldehyde at 4 °C, dehydrated in serial ethanol, embedded in paraffin, and cut into sections. The fibrosis in tissues was examined using a Masson’s trichrome staining kit (Solarbio Science & Technology Co., Ltd., Beijing, China). Apoptosis rate of cardiomyocytes in tissues was examined using a Terminal deoxynucleotidyl transferase (TdT)-mediated dUTP nick end labeling (TUNEL) kit. In addition, the expression of TGFBR2 (1:50, ab61213, Abcam) and p-Smad2 (phospho S255, ab188334, Abcam) in the myocardial tissues nearby the infarcted area was examined by immunohistochemical (IHC) staining. After staining, the sections were observed under an Axiophot light microscope (Zeiss Inc, AG, Oberkochen, Germany) and photographed by the connected digital camera. Quantitative analysis was analyzed by the Image J software. The fibrosis rate was calculated according to the ratio of fibrotic area to the total area of the field of view, while the apoptosis rate was calculated according to the ratio of TUNEL-positive cells to total cells in each field of view.

### Statistical Analysis

GraphPad Prism 8 (GraphPad, La Jolla, CA, USA) was used for data analysis. Data were collected from three experiments and exhibited as mean ± standard error of mean (SEM). Differences were analyzed by the unpaired *t* test (two groups) or one-way or two-way analysis of variance (ANOVA) followed by Tukey’s multiple test (multiple groups). *p < 0.05 represents statistical significance.

## RESULTS

### Identification of the adMSCs and the Exo

**Fig. 1:**
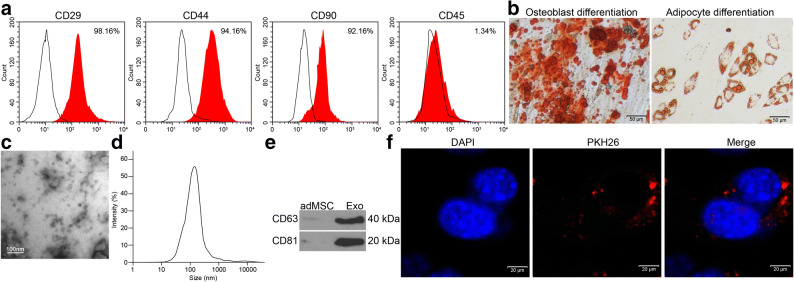
Identification of the adMSCs and the Exo. **a** Expression of the MSC-surface positive marker proteins CD29, CD44, and CD99 and the negative marker protein CD45 in the acquired adMSCs examined by flow cytometry; **b** Osteogenic differentiation and adipogenic differentiation potentials of the adMSCs examined by alizarin red staining and Oil red O staining, respectively; **c** Morphology of the extracted particles observed under the TEM; **d** Particle size distribution examined by a NTA; **e** Expression of the exosome surface marker proteins determined by western blot analysis; **f** Uptake of exosomes by cardiomyocytes observed by the PHK26 labeling. Three independent experiments were performed. Representative images are presented.

First, the flow cytometry confirmed positive expression of the MSC surface biomarkers CD29, CD44, and CD99 while negative expression of CD45 in the acquired adMSCs (Fig. 1a). The subsequent alizarin red staining and Oil red O staining results confirmed the ability of the adMSCs in differentiating into osteoblasts and adipoblasts, respectively (Fig. 1b). These results suggested that the adMSCs were qualified for further use.

The particles extracted from adMSCs were identified as well. Under the TEM, the particles were observed in a typical oval shape (Fig. 1c). The NTA results suggested that the size of the extracted particles was mainly distributed in 50–120 nm (Fig. 1d). The subsequent western blot assay confirmed positive expression of exosome-specific biomarkers CD63 and CD81 in the extracted particles (Fig. 1e). Collectively, these results showed that the particles extracted from adMSCs were exosomes. Thereafter, to examine whether these Exo can be internalized by the cells, an exosome uptake assay was performed. After 24 h of incubation, it was found that the PHK26-labeled Exo were obviously internalized by the cardiomyocytes (Fig. 1f).

### Exo Treatment Protects Cardiomyocytes Against OGD-Induced Damage

**Fig. 2:**
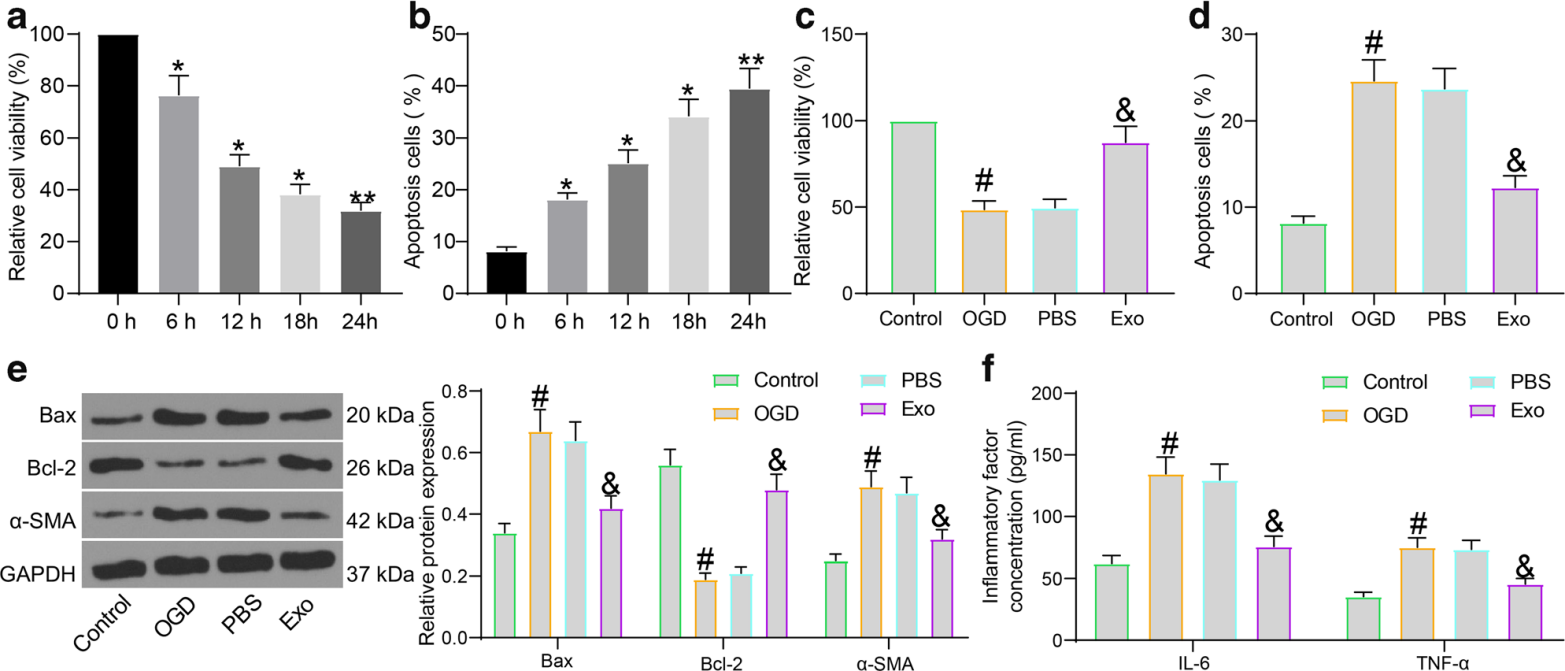
Exo treatment protects cardiomyocytes against OGD-induced damage. **a** Viability of cardiomyocytes at different time points after OGD treatment determined by the MTT assay; **b** Apoptosis of cardiomyocytes at different time points after OGD treatment examined by flow cytometry; **c** Viability of cardiomyocytes after Exo treatment examined by the MTT assay; **d** Apoptosis of cardiomyocytes after Exo treatment detected by flow cytometry; **e** Protein levels of apoptosis-related factors (Bax and Bcl-2) and the fibrosis-related factor α-SMA in OGD- and Exo-treated cardiomyocytes quantified by western blot analysis; **f** Production of pro-inflammatory cytokines (IL-6 and TNF-α) in OGD- and Exo-treated cardiomyocytes determined using ELISA kits. Data were collected from three experiments and exhibited as mean ± SEM. Differences were analyzed by one-way ANOVA (**a**–**d**) or two-way ANOVA (**e**, **f**); *p < 0.05, **p < 0.01 vs. 0 h; #*p* < 0.05 vs. control group; &*p* < 0.05 vs. PBS group.

Mouse cardiomyocytes were treated with OGD to mimic an MI condition *in vitro*. The MTT assay suggested that the viability of cells was gradually weakened in the process of OGD treatment. After 12 h, the viability of the cardiomyocytes reduced over a half (Fig. 2a). The apoptosis rate of the cardiomyocytes was examined by flow cytometry. As expected, the number of apoptotic cardiomyocytes was increased in the process of OGD treatment. After 12 h, the apoptosis rate of cells was increased by two times compared to that at 0 h (Fig. 2b).

Next, the cardiomyocytes underwent 12 h of OGD treatment were then co-cultured with Exo. Those further treated with an equal volume of PBS. According to the MTT assay, the viability of cardiomyocytes was significantly suppressed by OGD treatment. Compared to PBS treatment, Exo treatment significantly restored the viability of cells (Fig. 2c). Also, the flow cytometry results showed that the OGD significantly enhanced the apoptosis of cardiomyocytes. Compared to PBS treatment, Exo treatment significantly reduced the apoptosis rate of cells (Fig. 2d).

Myocardial fibrosis and inflammation are key factors affecting MI prognosis [22, 23]. Here, we further examined the expression of apoptosis-related molecules Bax and Bcl-2 and the expression of the fibrosis biomarker α-SMA in cells. The western blot analysis suggested that OGD treatment increased the expression of α-SMA and the pro-apoptotic Bax while reduced the expression of anti-apoptotic Bcl-2. Importantly, the expression of Bax and α-SMA was reduced while the expression of Bcl-2 was increased by Exo (Fig. 2e). The protein levels of IL-6 and TNF-α in cells were examined using ELISA kits to evaluate the inflammatory response in cells. It was found that OGD led to a notable increase in the secretion of pro-inflammatory cytokines IL-6 and TNF-α in the cardiomyocytes. Again, treatment of Exo reduced the secretion of IL-6 and TNF-α in cells (Fig. 2f). These results, collectively, confirmed the protective roles of adMSC-derived exosomes in OGD-treated cardiomyocytes.

### Exo Treatment Enhances miR-671 Expression in Cardiomyocytes

**Fig. 3:**
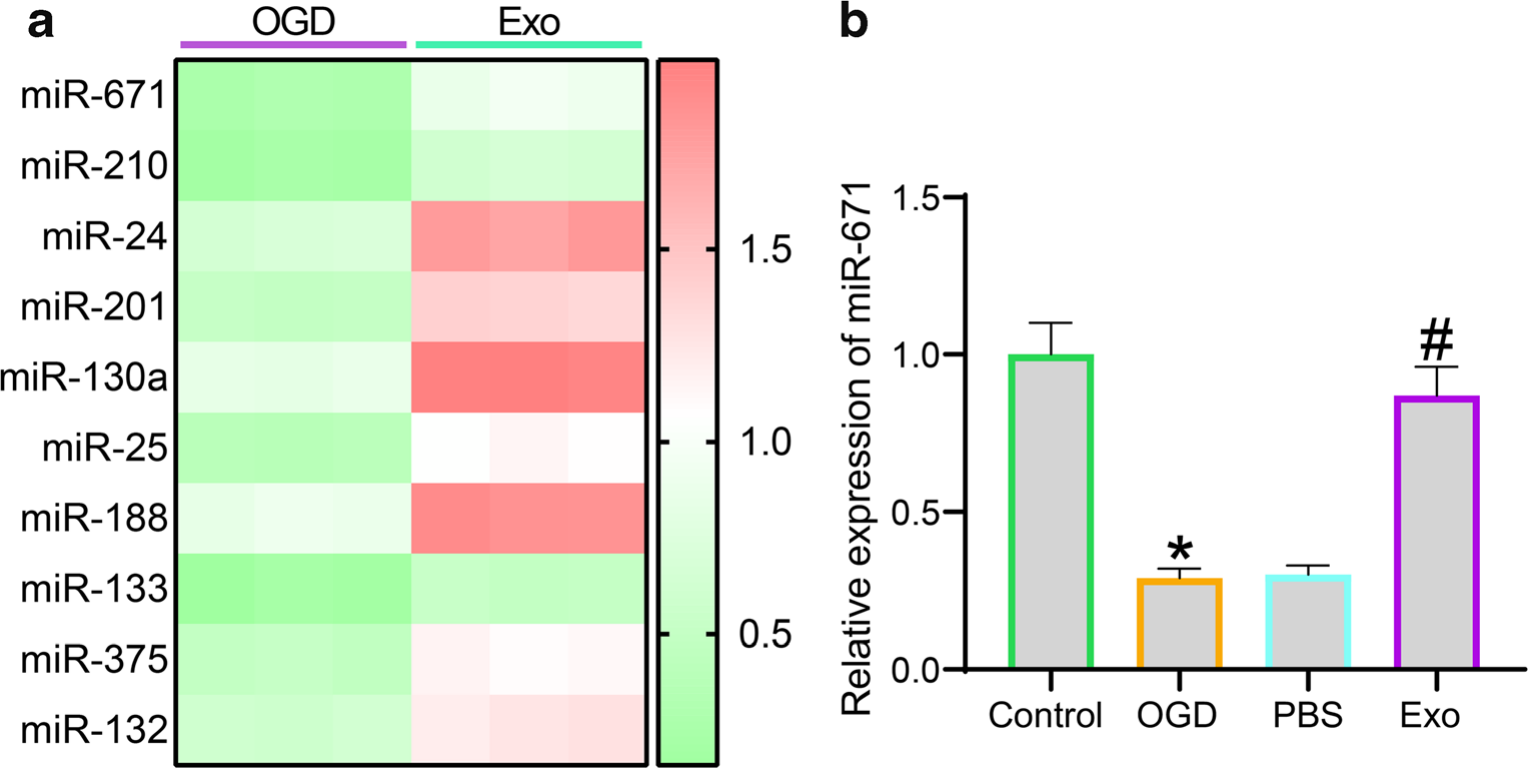
Exo treatment enhances miR-671 expression in cardiomyocytes. **a** Top 10 differentially expressed miRNAs in cardiomyocytes before and after Exo treatment using a miRNA microarray analysis; **b** Expression of miR-671 in cardiomyocytes after OGD and Exo treatment determined by RT-qPCR. Data were collected from three experiments and exhibited as mean ± SEM. Differences were analyzed by one-way ANOVA (**b**); *p < 0.05 vs. control group; #*p* < 0.05 vs. PBS group.

As discussed above, exosomes frequently exert their functions through the delivery of miRNAs. Thereby, we examined the differentially expressed miRNAs in cardiomyocytes before and after Exo treatment using a miRNA microarray analysis. Using the fold change ≥ 2 as the threshold, 10 mostly changed miRNAs in cardiomyocytes after Exo treatment are presented in Fig. 3a, among which miR-671 showed the highest degree of upregulation (fold change ≈ 3). Then, the RT-qPCR results showed that the miR-671 expression in cardiomyocytes was initially decreased after OGD treatment but then restored after further Exo treatment (Fig. 3b).

### Downregulation of miR-671 Blocks the Protective Functions of Exo on Cardiomyocytes

**Fig. 4:**
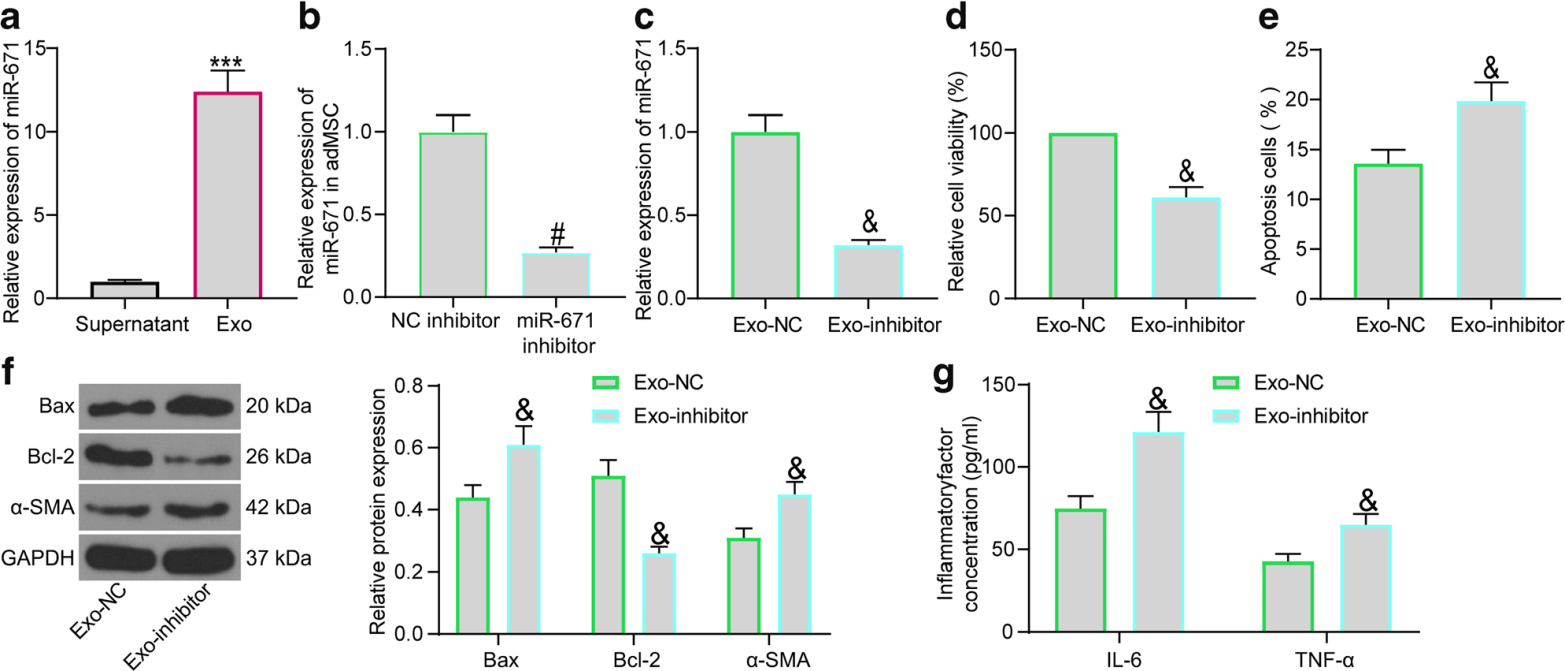
Downregulation of miR-671 blocks the protective functions of Exo on cardiomyocytes. **a** miR-671 expression in the exosome precipitates and the supernatant after centrifugation using RT-qPCR; **b** Transfection efficiency of miR-671 inhibitor in adMSCs examined by RT-qPCR; **c** miR-671 expression in the exosomes determined by RT-qPCR; **d** Viability of OGD-cardiomyocytes after Exo-NC or Exo-inhibitor treatment determined by the MTT assay; **e** Apoptosis of OGD-cardiomyocytes after Exo-NC or Exo-inhibitor treatment examined by flow cytometry; **f** Protein levels of apoptosis-related factors (Bax and Bcl-2) and the fibrosis-related factor α-SMA in OGD-cardiomyocytes after Exo-NC or Exo-inhibitor treatment determined by western blot analysis; **g** Production of pro-inflammatory cytokines (IL-6 and TNF-α) in OGD-cardiomyocytes after Exo-NC or Exo-inhibitor treatment measured using ELISA kits. Data were collected from three experiments and exhibited as mean ± SEM. Differences were analyzed by unpaired *t* test (**a**–**e**) or two-way ANOVA (**f**, **g**); ***p < 0.001 vs. supernatant; #*p* < 0.05 vs. NC inhibitor group; &*p* < 0.05 vs. Exo-NC group.

To validate whether miR-671 is responsible for the protective events mediated by the Exo, we first examined the abundancy of miR-671 in the exosome precipitates and the supernatant after centrifugation. Consequently, an enrichment of miR-671 was confirmed in the precipitated particles (Fig. 4a). Thereafter, interfering experiments were performed through administration of miR-671 inhibitor or the NC-inhibitor in the adMSCs, and the successful transfection was examined by RT-qPCR (Fig. 4b). Then, the exosomes extracted from these adMSCs were collected, named Exo-NC and Exo-inhibitor, correspondingly. Compared to Exo-NC, the Exo-inhibitor were found to have significantly reduced level of miR-671(Fig. 4c).

The OGD-treated cardiomyocytes were then treated with Exo-NC or Exo-inhibitor. In this setting, the MTT assay suggested that Exo-inhibitor reduced the viability of cardiomyocytes compared to Exo-NC (Fig. 4d). Then, the apoptosis rate of cells was examined by flow cytometry again. It was found that downregulation of miR-671 in Exo led to a notable increase in the number of apoptotic cardiomyocytes (Fig. 4e). The above results indicated that Exo treatment decreased the expression of α-SMA and Bax while increased Bcl-2 expression in cells, while downregulation of miR-671 in Exo-inhibitor led to inverse trends (Fig. 4f). The ELISA results also found that the suppressive function of the Exo on the release of pro-inflammatory cytokines in cardiomyocytes was blocked upon miR-671 inhibition (Fig. 4g).

### miR-671 Directly Targets TGFBR2

**Fig. 5:**
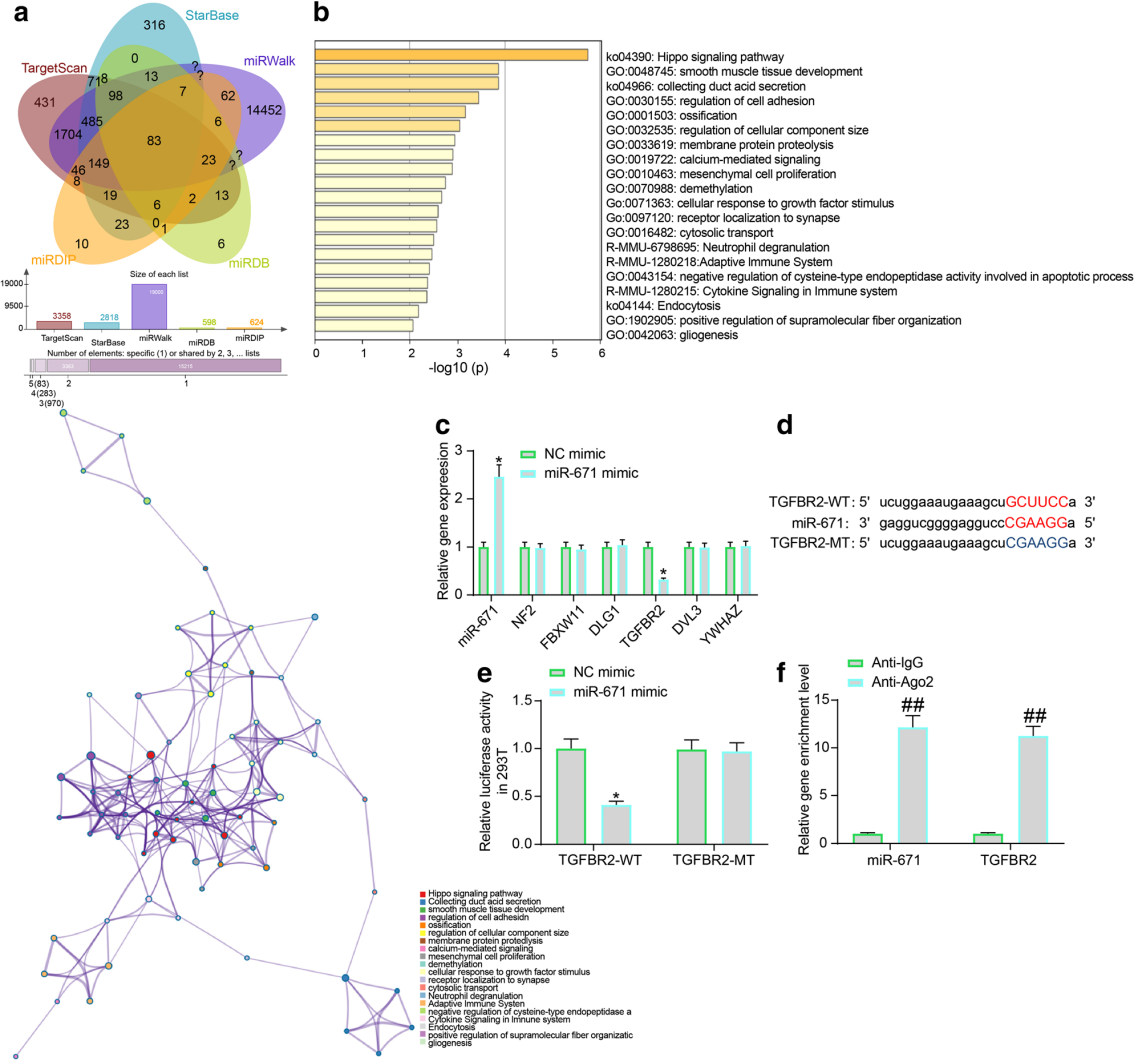
miR-671 directly targets TGFBR2. **a** Putative target transcripts of miR-671 predicted using five bioinformatic systems; **b** A KEGG pathway enrichment analysis based on the above predicted mRNAs; **c** Expression of miR-671 and the mRNA expression of NF2, FBXW11, DLG, TGFBR2, DVL3, and YWHAZ in mouse cardiomyocytes after miR-671 mimic transfection examined by RT-qPCR; **d** Putative binding sequence between TGFBR2 and miR-671 and the mutant binding sequence for the construction of luciferase vectors; **e** Binding relationship between TGFBR2 and miR-671 validated through a dual luciferase assay; **f** Enrichment of miR-671 and TGFBR2 fragments in the compounds pulled down by anti-Ago2 examined by the RIP assay. Data were collected from three experiments and exhibited as mean ± SEM. Differences were analyzed by two-way ANOVA (**c**, **e**, and **f**); *p < 0.05 vs. NC mimic group; ##*p* < 0.01 vs. anti-IgG.

To identify the downstream molecules mediated by miR-671, we then predicted the possible target transcripts of miR-671 using several bioinformatic systems including TargetScan (http://www.targetscan.org/vert_72/), StarBase, miRwalk (http://mirwalk.umm.uni-heidelberg.de/), miRDB (http://mirdb.org/), and miRDIP (http://ophid.utoronto.ca/mirDIP/), and 83 common outcomes were obtained (Fig. 5a). Then, a Kyoto Encyclopedia of Genes and Genomes (KEGG) enrichment analysis was performed based on these genes (Fig. 5b), and six key target genes including NF2, FBXW11, DLG, TGFBR2, DVL3, and YWHAZ were identified.

Then, miR-671 mimic and the NC mimic were transfected into the mouse cardiomyocytes, and then the expression of miR-671 and the mRNA expression of the above six genes was determined by RT-qPCR (Fig. 5c). It was found that miR-671 mimic enhanced the expression of miR-671 in cells and reduced the expression of TGFBR2. However, the expression of the reminding five candidate genes showed little change. Next, the putative binding site between miR-671 and TGFBR2 was obtained from StarBase to construct the TGFBR2-WT/TGFBR2-MT vectors (Fig. 5d). The vectors were co-transfected with miR-671 mimic or NC mimic into 293T cells. The miR-671 mimic was found to specifically suppressed the luciferase activity of TGFBR2-WT in cells (Fig. 5e). In addition, the subsequent RIP assay further confirmed that the miR-671 and TGFBR2 fragments were enriched by anti-Ago2 compared to anti-IgG (Fig. 5f).

### Exosomal miR-671 Targets TGFBR2 and Reduces Smad2 Phosphorylation

**Fig. 6:**
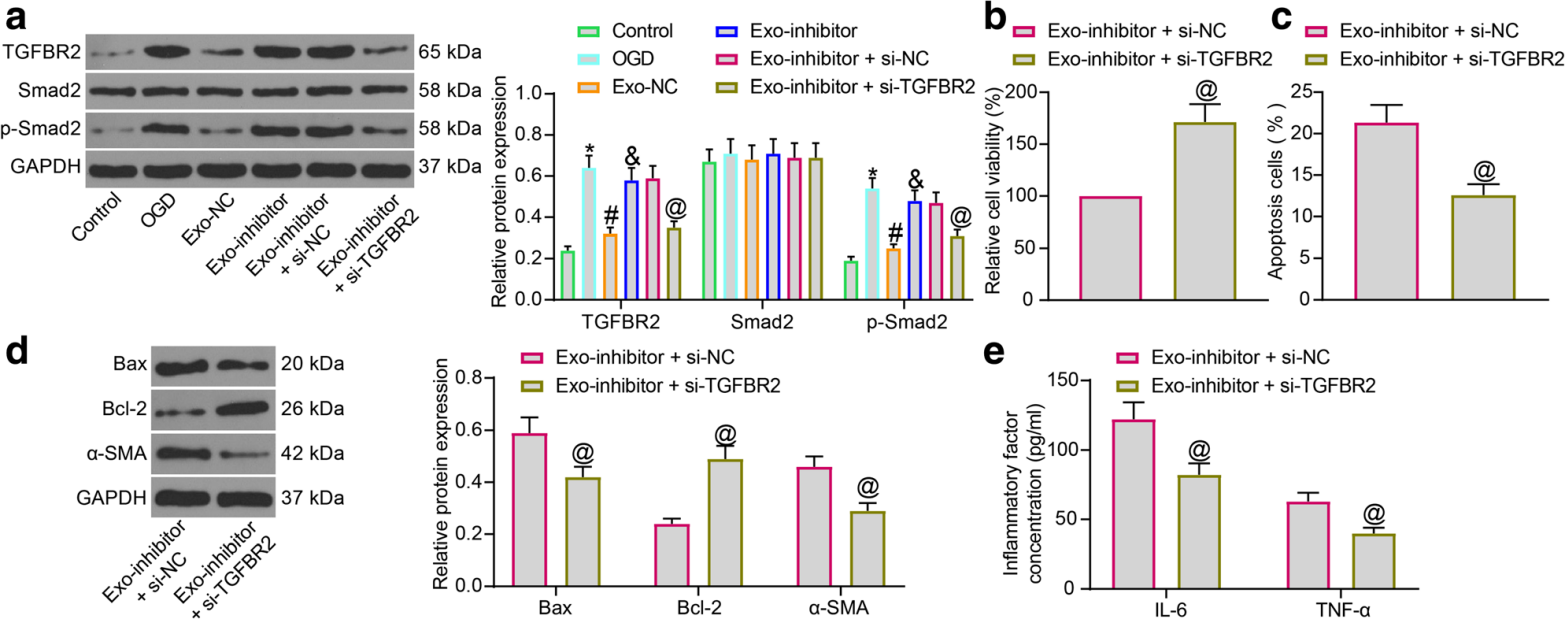
Exosomal miR-671 targets TGFBR2 and reduces Smad2 phosphorylation. **a** Protein levels of TGFBR2, Smad2 and the phosphorylation of Smad2 in each group of cardiomyocytes examined by western blot analysis; **b** Viability of cardiomyocytes after si-TGFBR2 transfection measured by the MTT assay; **c** Apoptosis of cardiomyocytes after si-TGFBR2 transfection examined by flow cytometry; **d** Protein levels of apoptosis-related factors (Bax and Bcl-2) and the fibrosis-related factor α-SMA in cardiomyocytes after si-TGFBR2 transfection determined by western blot analysis; **e** Production of pro-inflammatory cytokines (IL-6 and TNF-α) in cardiomyocytes after si-TGFBR2 transfection determined using ELISA kits. Data were collected from three experiments and exhibited as mean ± SEM. Differences were analyzed by unpaired *t* test (**b**, **c**) or two-way ANOVA (**a**, **d**, and **e**); *p < 0.05 vs. Control group; #*p* < 0.05 vs. OGD group; &*p* < 0.05 vs. Exo-NC group; @*p* <0.05 vs. Exo-inhibitor + si-NC group.

As mentioned before, TGFBR2 is a key regulator activating phosphorylation of Smad2. Activation of the Smad2 has been implicated in the pathogenesis of MI [24]. Here, the cardiomyocytes in the Exo-inhibitor group were further transfected with si-TGFBR2 or si-NC, and then the protein levels of TGFBR2, Smad2 and the phosphorylation of Smad2 in each group of cardiomyocytes were determined by western blot assays. It was found that OGD treatment significantly enhanced the level of TGFBR2 protein as well as the Smad2 activation in cells, while Exo-NC suppressed the protein level of TGFBR2 as well as the phosphorylation of Smad2. Again, these suppressive functions of Exo were diminished after miR-671 knockdown. Further administration of si-TGFBR2 reduced the expression of TGFBR2 and reduced phosphorylation of Smad2 in cells (Fig. 6a).

We then examined the viability of cardiomyocytes in the Exo-inhibitor group after further si-TGFBR2 or si-NC transfection. The MTT assay suggested that downregulation of TGFBR2 restored the viability of cardiomyocytes (Fig. 6b). As expected, the flow cytometry results showed that downregulation of TGFBR2 reduced the apoptosis rate of cardiomyocytes (Fig. 6c). The western blot assay suggested that downregulation of TGFBR2 suppressed the expression of Bax and α-SMA and enhanced the expression of Bcl-2 in cardiomyocytes (Fig. 6d). In addition, the ELISA assay results suggested that si-TGFBR2 also suppressed the secretion of inflammatory cytokines including IL-6 and TNF-α in cells (Fig. 6e).

### Exosomal miR-671 Alleviates Fibrosis and Cell Apoptosis in Myocardial Tissues in Mice with MI

**Fig. 7:**
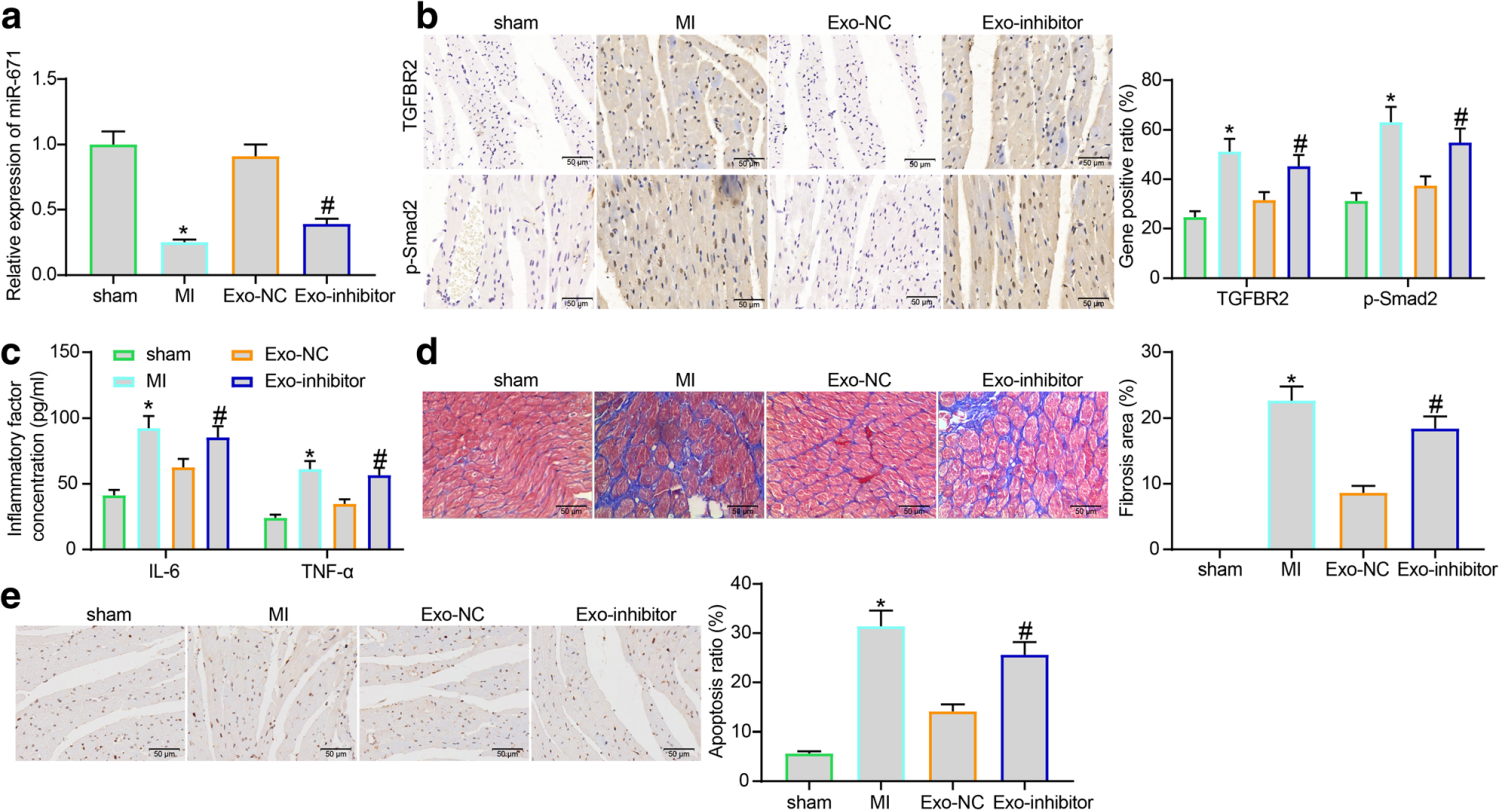
Exosomal miR-671 alleviates fibrosis and cell apoptosis in myocardial tissues in mice with MI. **a** miR-671 expression in murine myocardial tissues nearby the infarcted area detected using RT-qPCR; **b** Protein levels of TGFBR2 and p-Smad2 in murine myocardial tissues nearby the infarcted area examined by IHC staining; **c** Concentrations of IL-6 and TNF-α in the homogenate of murine myocardial tissues examined by ELISA kits; **d** Myocardial fibrosis in mouse examined by masson's trichrome staining; **e** Cell apoptosis in murine myocardial tissues examined by TUNEL assay. *N* = 8 in each group; representative images are provided. Data were collected from three experiments and exhibited as mean ± SEM. Differences were analyzed by one-way ANOVA (**a**, **d**, and **e**) or two-way ANOVA (**b**, **c**); *p < 0.05 vs. sham group; #*p* < 0.05 vs. Exo-NC group.

A murine model of MI was induced, and the model mice were treated with Exo-NC or Exo-inhibitor. First, we examined the miR-671 expression in myocardial tissues nearby the infarcted area using RT-qPCR. Compared to the sham-operated ones, the mice in the MI group owned decreased expression of miR-671. Likewise, the expression of miR-671 was increased in mice after Exo-NC treatment but decreased after Exo-inhibitor treatment (Fig. 7a).

Then, the expression of TGFBR2 and p-Smad2 in the murine myocardial tissues was examined by IHC staining. The staining intensity of TGFBR2 and p-Smad2 in the myocardial tissues in model mice was significantly enhanced. Further administration of Exo-NC reduced the protein levels of TGFBR2 and p-Smad2, while this reduction was blocked in the Exo-inhibitor group where miR-671 expression was weakened (Fig. 7b). In addition, the concentrations of IL-6 and TNF-α in the MI model mice were reduced by Exo-NC but elevated by Exo-inhibitor. Again, the inflammatory response in murine myocardial tissues was alleviated by Exo-NC, while the anti-inflammatory function was significantly weakened in Exo-inhibitor (Fig. 7c).

Furthermore, Masson’s trichrome staining suggested that Exo-NC reduced the myocardial fibrosis in the model mice with MI. However, the anti-fibrotic function of the exosomes was blocked again when miR-671 was suppressed (Fig. 7d). The TUNEL assay further confirmed increased cell apoptosis in the myocardial tissues in model mice. Treatment of Exo-NC alleviated cell apoptosis *in vivo* as well. Still, when the expression of miR-671 was inhibited, the anti-apoptotic effect of the Exo was notably weakened (Fig. 7e). These results indicated that miR-671 is at least partially accountable for the myocardial-protective roles of the Exo *in vivo*.

## DISCUSSION

MI remains a major contributor to ischemic heart disease that represents a significant cause of morbidity and mortality across the global. Cellular therapy has seen promises and possibilities in the treatment for MI-induced ischemic heart disease, especially typical MSCs and their derived extracellular vesicles including exosomes [25]. In this study, we confirmed that Exo showed potent anti-apoptotic, anti-inflammatory, and anti-fibrotic properties *in vitro* and *in vivo* through the delivery of miR-671 and the subsequent suppression of the TGFBR2/Smad2 axis.

The broad distribution of sources containing MSCs along the capacity of MSCs in differentiating into many mesenchymal phenotypes allows them as potential therapeutic candidates for multiple diseases including cardiac diseases and MI [26, 27]. MSC injection in the infarct area in a rat model with MI decreased the infarct size significantly [28]. The mediation of MSCs on the functions of surrounding cells such as cardiomyocyte, epithelial cells, and neurons has been reported as well [29, 30]. However, it is currently well-recognized that beneficial functions of MSCs are transient and achieved through the release of soluble paracrine factors and extracellular vesicles including exosomes [31–34]. Emerging evidence suggested the beneficial cardio-protective roles of MSC-derived exosomes in reducing cell death and fibrosis [35, 36]. Here, we observed that Exo treatment enhanced the viability of OGD-treated cardiomyocytes and reduced the cell apoptosis rate as well. In a molecular perspective, the Exo treatment reduced the expression of pro-apoptotic Bax and fibrotic marker protein α-SMA, but it elevated the expression of anti-apoptotic Bcl-2 in the OGD-treated cells. Reduced cell apoptosis, myocardial fibrosis, and inflammation were reproduced *in vivo* in a murine model with MI. These results collectively confirmed a cardio-protective role of the Exo.

MiRNAs are a major type of exosome cargos and are undoubtedly involved in almost every facet of repair mechanisms of MSC-based therapy in MI, including stem cell differentiation, apoptosis, neovascularization, cardiac remodeling, arrhythmias, and cardiac contractility and so forth [37]. For instance, localized injection of miRNA-21-abundant extracellular vesicles enhanced cardiac function following MI [6]. Upregulation of miR-133 has been documented to enhance the therapeutic efficacy of MSCs on acute MI [38]. In the present research, a miRNA microarray analysis was performed to analyze the differentially expressed miRNAs in cardiomyocytes after Exo treatment, and miR-671 was identified as the miRNA with the highest degree of upregulation. Although its direct correlation with cardiac protection has been rarely concerned, miR-671 has been reported to be poorly expressed in patients with coronary artery disease [12]. In addition, exosomal miR-671 has been suggested as a biomarker for the diagnosis of Kawasaki disease whose upregulation reduced vascular inflammation [39]. This miRNA has also shown anti-inflammatory functions in osteoarthritis both *in vitro* and *in vivo* [40]. In order to confirm the involvement of miR-671 in the Exo-mediated events, loss-of-function studies were performed by suppressing miR-671 expression in the adMSCs, and then the protective effects of the Exo were significantly blocked. Downregulation of miR-671 in exosomes increased cell apoptosis, inflammation, and myocardial fibrosis in OGD-treated cardiomyocytes and the model mice with MI. Therefore, we reported that miR-671 has a cardio-protective role in MI and it is at least partially responsible for the events mediated by MSC and the exosomes.

It is reported that over 30% of protein-coding genes in human genome can be regulated by miRNAs, and they are inevitably implicated in the cascades mediated by miRNAs [41]. In our study, the integrated microarray analysis and cellular experiments suggested that miR-671 targets TGFBR2, an important receptor protein of the TGF-β signaling pathway. As aforementioned, TGF-β activates the TGFBRs to regulate the downstream Smad family. TGFBR2 has been reported as a positive regulator of Smad2 in several pathological conditions [17, 18, 42, 43]. Also, the TGF-β/Smad2 pathway has been recognized as a traditional pathway which initiates activation of cardiac fibroblasts [44]. Targeting the TGF-β/JNK axis has also been validated to attenuate myocardial cell apoptosis in a rat model in the setting of MI [45]. Likewise, increased phosphorylation of Smad2 has been reported to be relevant to increased cardiomyocyte apoptosis, myocardial inflammation, and fibrosis [24, 46, 47]. Here, our study evidenced that the Exo reduced the expression of TGFBR2 as well as the Smad2 phosphorylation in OGD-treated cells. As expected, downregulation of miR-671 in exosomes reactivated the TGFBR2/Smad2 axis in cells. The additional loss-of-function assays found that downregulation of TFGBR2 also enhanced the viability while reduced apoptosis, fibrosis, and inflammation in cells. Similarly, a recent study by Yuan Lin *et al.* suggested that MSC-derived exosomes improved myocardial injury and fibrosis induced by diabetes mellitus through the inhibition of TGF-β1/Smad2 signaling pathway [48].

## CONCLUSION

**Fig. 8:**
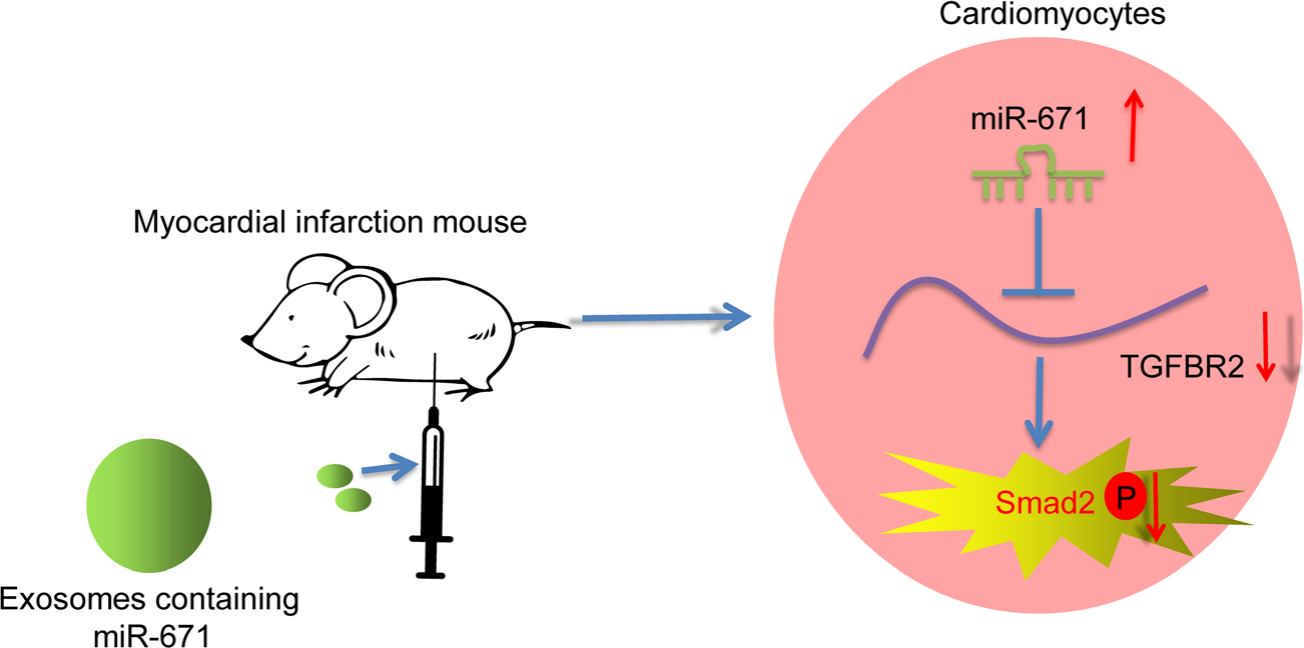
A diagram for the molecular mechanism. AdMSC-derived exosomal miR-671 directly targets the TGFBR2/Smad2 axis, therefore alleviating OGD-induced damage on mouse cardiomyocytes *in vitro* and myocardial injury in model mice with MI.

To conclude, this study evidenced that adMSC-derived exosomal miR-671 directly targets the TGFBR2/Smad2 axis and alleviates OGD-induced damage on mouse cardiomyocytes *in vitro* and myocardial injury in model mice with MI (Fig. 8). This study validated the protective functions of Exo on myocardium and provided novel evidence for the possible clinical application of MSCs and the exosomes on MI management. We also hope more studies in this field will be conducted to provide more insights into the treatment of MI and heart diseases.

## Data Availability

The datasets used and/or analyzed during the current study are available from the corresponding author on reasonable request.
